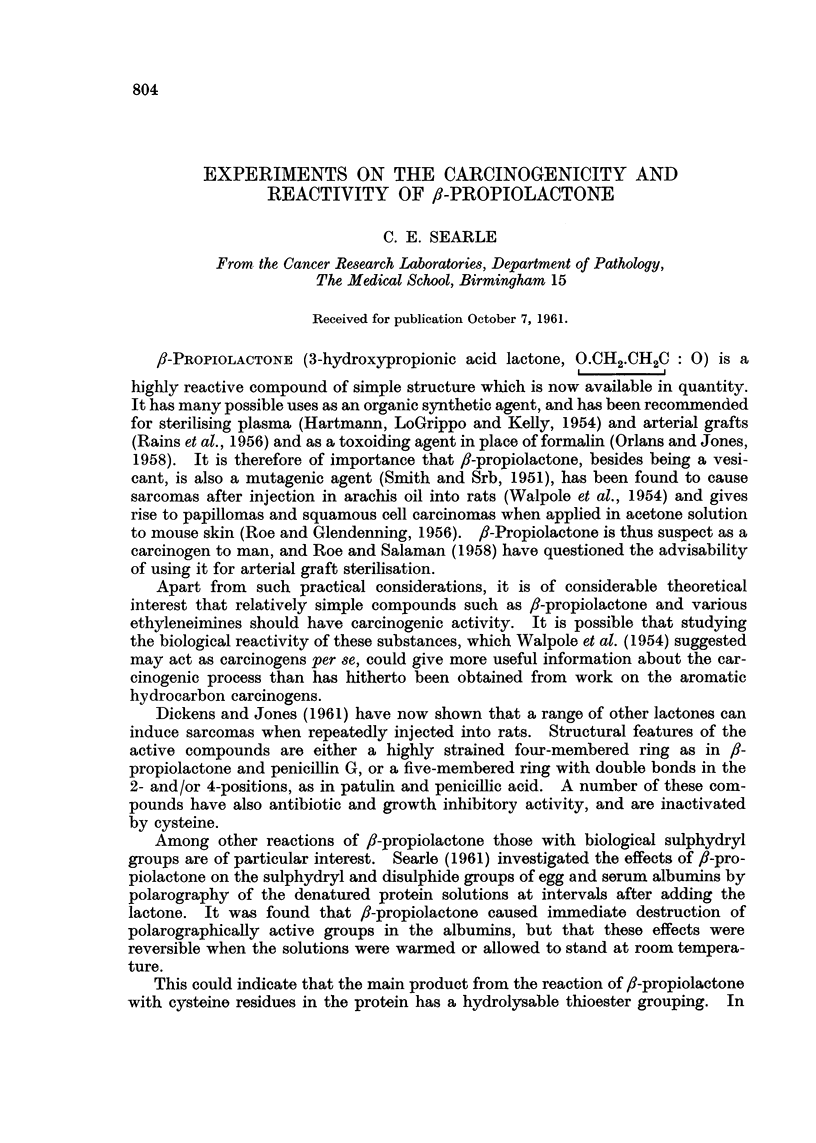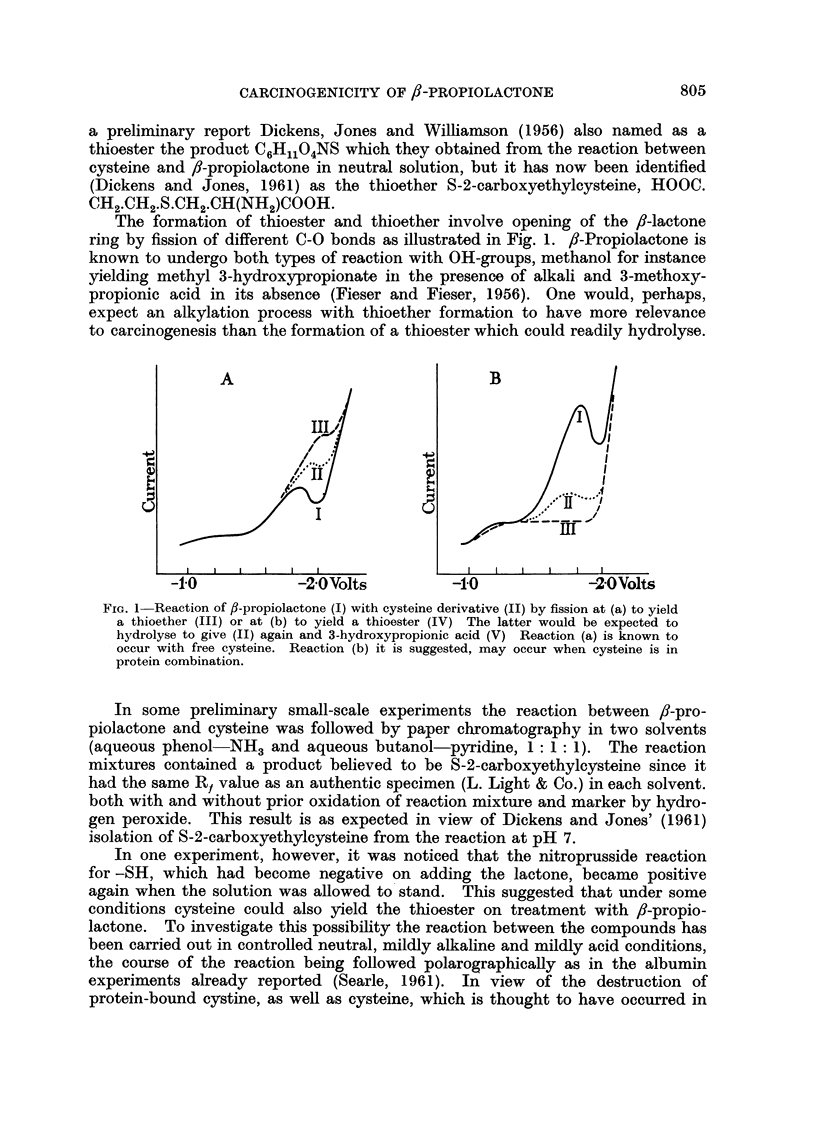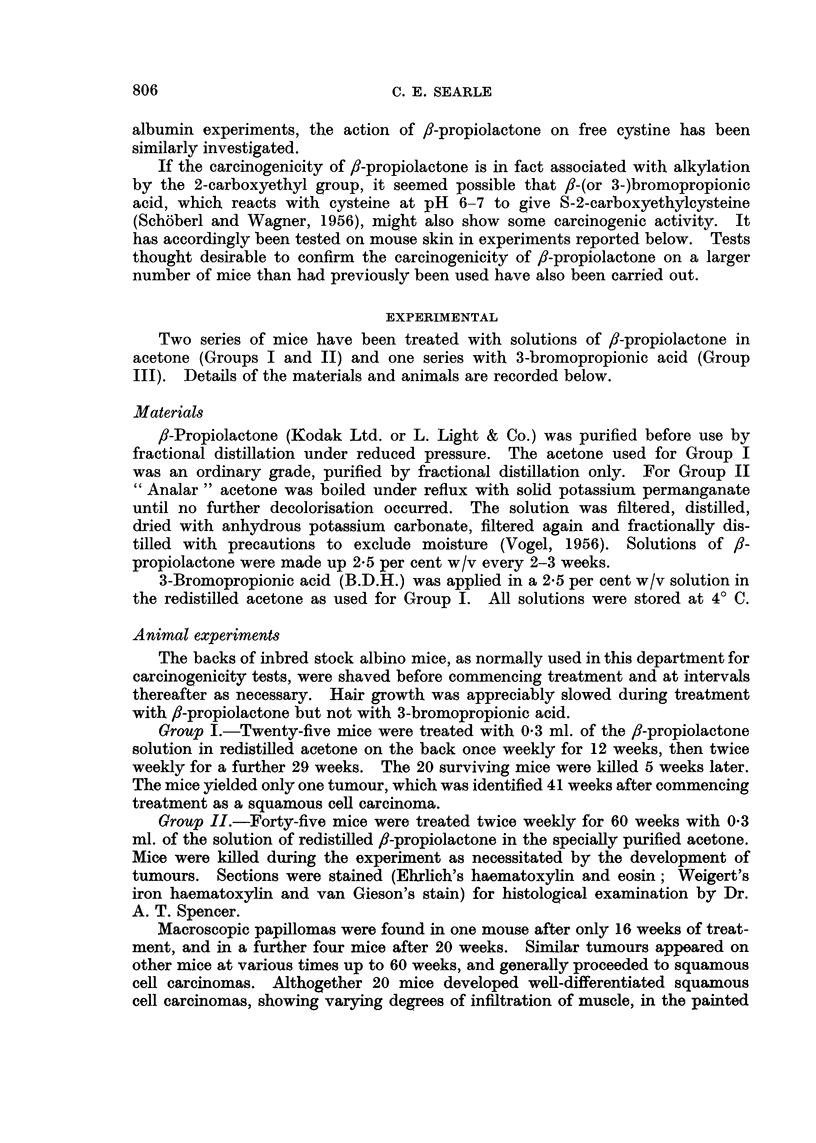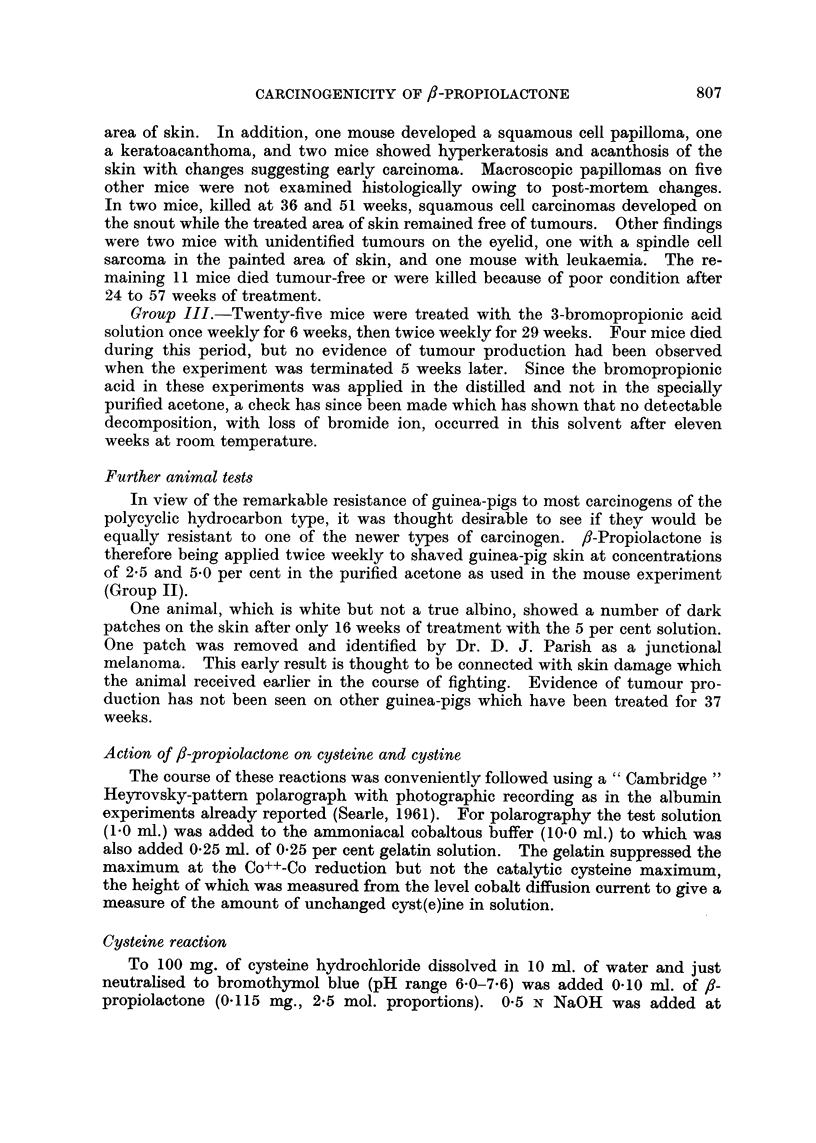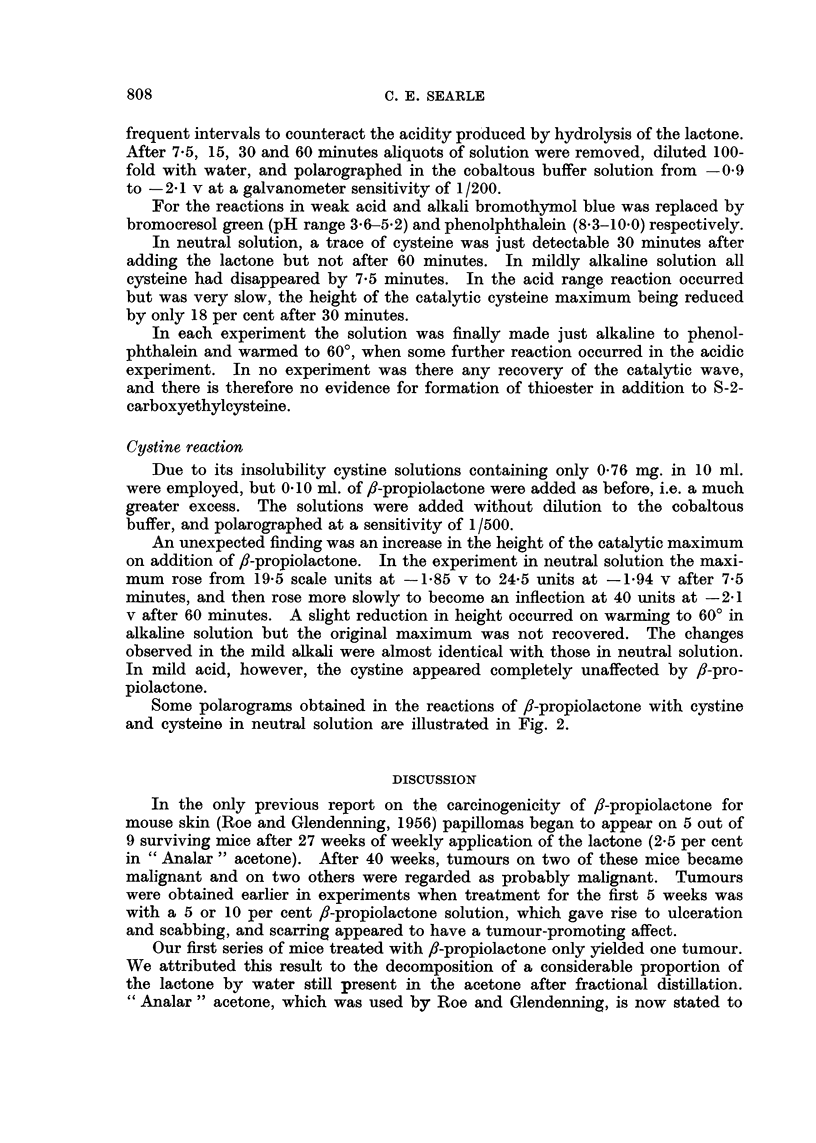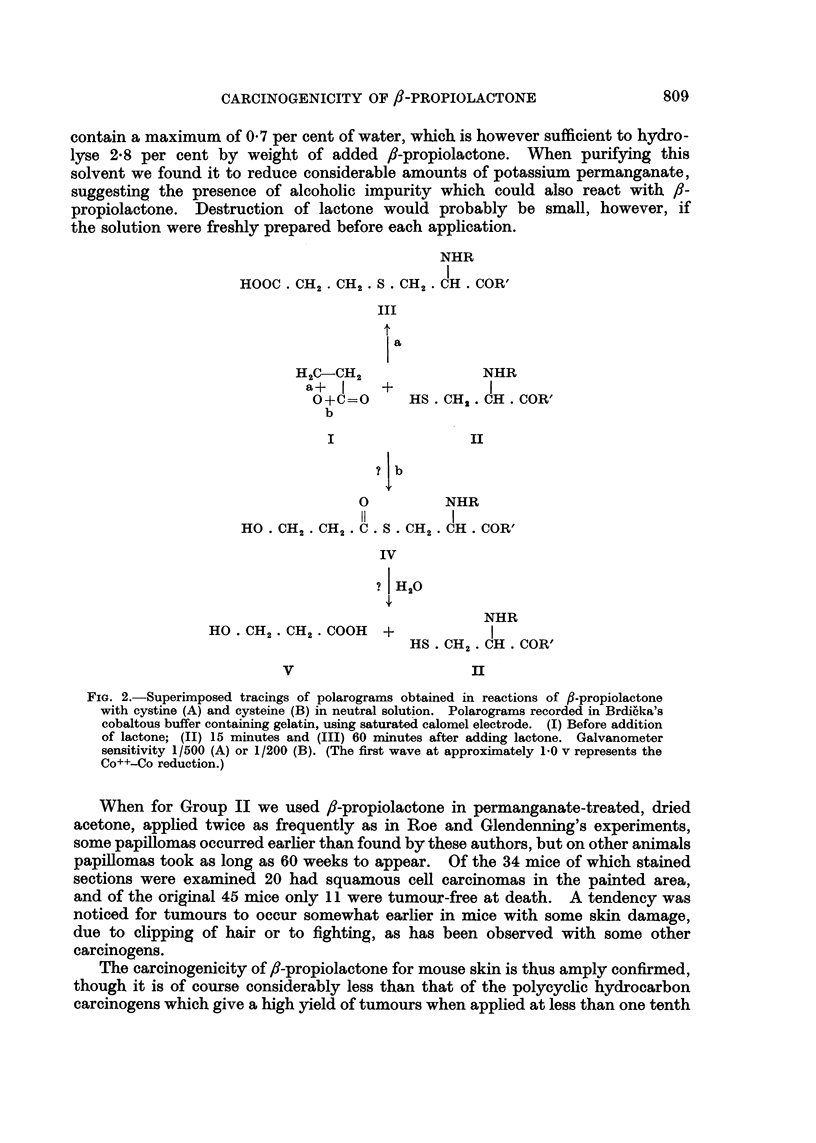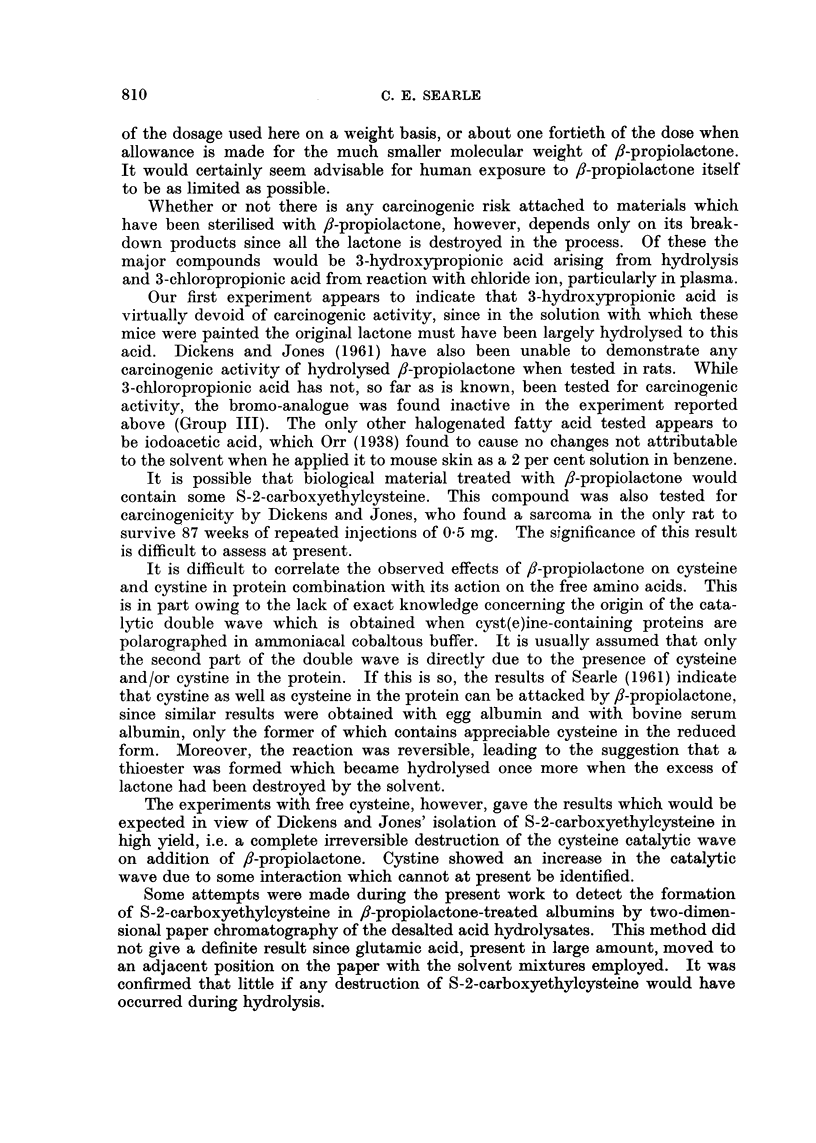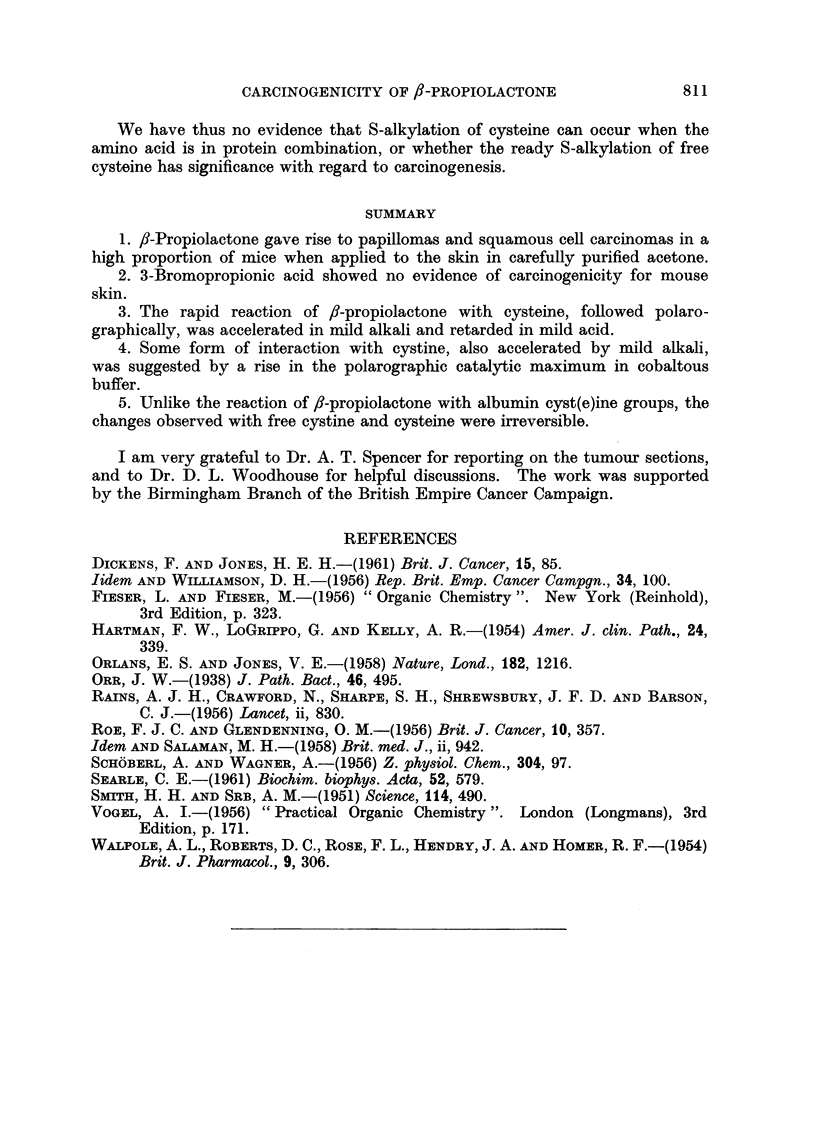# Experiments on the Carcinogenicity and Reactivity of β-propiolactone

**DOI:** 10.1038/bjc.1961.91

**Published:** 1961-12

**Authors:** C. E. Searle


### EXPERIMENTS ON THE CARCINOGENICITY AND REACTIVITY OF β-PROPIOLACTONE

C. E. SEARLE

*From the Cancer Research Laboratories, Department of Pathology, The Medical School, Birmingham* 15

Received for publication October 7, 1961.

β-PROPIOLACTONE (3-hydroxypropionic acid lactone, $\underline{O.CH_2.CH_2C}$ : O) is a highly reactive compound of simple structure which is now available in quantity. It has many possible uses as an organic synthetic agent, and has been recommended for sterilising plasma (Hartmann, LoGrippo and Kelly, 1954) and arterial grafts (Rains *et al.*, 1956) and as a toxoiding agent in place of formalin (Orlans and Jones, 1958). It is therefore of importance that β-propiolactone, besides being a vesicant, is also a mutagenic agent (Smith and Srb, 1951), has been found to cause sarcomas after injection in arachis oil into rats (Walpole *et al.*, 1954) and gives rise to papillomas and squamous cell carcinomas when applied in acetone solution to mouse skin (Roe and Glendenning, 1956). β-Propiolactone is thus suspect as a carcinogen to man, and Roe and Salaman (1958) have questioned the advisability of using it for arterial graft sterilisation.

Apart from such practical considerations, it is of considerable theoretical interest that relatively simple compounds such as β-propiolactone and various ethyleneimines should have carcinogenic activity. It is possible that studying the biological reactivity of these substances, which Walpole *et al.* (1954) suggested may act as carcinogens *per se*, could give more useful information about the carcinogenic process than has hitherto been obtained from work on the aromatic hydrocarbon carcinogens.

Dickens and Jones (1961) have now shown that a range of other lactones can induce sarcomas when repeatedly injected into rats. Structural features of the active compounds are either a highly strained four-membered ring as in β-propiolactone and penicillin G, or a five-membered ring with double bonds in the 2- and/or 4-positions, as in patulin and penicillic acid. A number of these compounds have also antibiotic and growth inhibitory activity, and are inactivated by cysteine.

Among other reactions of β-propiolactone those with biological sulphydryl groups are of particular interest. Searle (1961) investigated the effects of β-propiolactone on the sulphydryl and disulphide groups of egg and serum albumins by polarography of the denatured protein solutions at intervals after adding the lactone. It was found that β-propiolactone caused immediate destruction of polarographically active groups in the albumins, but that these effects were reversible when the solutions were warmed or allowed to stand at room temperature.

This could indicate that the main product from the reaction of β-propiolactone with cysteine residues in the protein has a hydrolysable thioester grouping. In a preliminary report Dickens, Jones and Williamson (1956) also named as a thioester the product $C_6H_{11}O_4NS$ which they obtained from the reaction between cysteine and β-propiolactone in neutral solution, but it has now been identified (Dickens and Jones, 1961) as the thioether S-2-carboxyethylcysteine, HOOC. $CH_2.CH_2.S.CH_2.CH(NH_2)COOH$.

The formation of thioester and thioether involve opening of the β-lactone ring by fission of different C-O bonds as illustrated in Fig. 1. β-Propiolactone is known to undergo both types of reaction with OH-groups, methanol for instance yielding methyl 3-hydroxypropionate in the presence of alkali and 3-methoxy-propionic acid in its absence (Fieser and Fieser, 1956). One would, perhaps, expect an alkylation process with thioether formation to have more relevance to carcinogenesis than the formation of a thioester which could readily hydrolyse.

FIG. 1—Reaction of β-propiolactone (I) with cysteine derivative (II) by fission at (a) to yield a thioether (III) or at (b) to yield a thioester (IV) The latter would be expected to hydrolyse to give (II) again and 3-hydroxypropionic acid (V) Reaction (a) is known to occur with free cysteine. Reaction (b) it is suggested, may occur when cysteine is in protein combination.

In some preliminary small-scale experiments the reaction between β-propiolactone and cysteine was followed by paper chromatography in two solvents (aqueous phenol—$NH_3$ and aqueous butanol—pyridine, 1 : 1 : 1). The reaction mixtures contained a product believed to be S-2-carboxyethylcysteine since it had the same $R_f$ value as an authentic specimen (L. Light & Co.) in each solvent. both with and without prior oxidation of reaction mixture and marker by hydrogen peroxide. This result is as expected in view of Dickens and Jones' (1961) isolation of S-2-carboxyethylcysteine from the reaction at pH 7.

In one experiment, however, it was noticed that the nitroprusside reaction for –SH, which had become negative on adding the lactone, became positive again when the solution was allowed to stand. This suggested that under some conditions cysteine could also yield the thioester on treatment with β-propiolactone. To investigate this possibility the reaction between the compounds has been carried out in controlled neutral, mildly alkaline and mildly acid conditions, the course of the reaction being followed polarographically as in the albumin experiments already reported (Searle, 1961). In view of the destruction of protein-bound cystine, as well as cysteine, which is thought to have occurred in albumin experiments, the action of $\beta$-propiolactone on free cystine has been similarly investigated.

If the carcinogenicity of $\beta$-propiolactone is in fact associated with alkylation by the 2-carboxyethyl group, it seemed possible that $\beta$-(or 3-)bromopropionic acid, which reacts with cysteine at pH 6–7 to give S-2-carboxyethylcysteine (Schöberl and Wagner, 1956), might also show some carcinogenic activity. It has accordingly been tested on mouse skin in experiments reported below. Tests thought desirable to confirm the carcinogenicity of $\beta$-propiolactone on a larger number of mice than had previously been used have also been carried out.

### EXPERIMENTAL

Two series of mice have been treated with solutions of $\beta$-propiolactone in acetone (Groups I and II) and one series with 3-bromopropionic acid (Group III). Details of the materials and animals are recorded below.

### *Materials*

$\beta$-Propiolactone (Kodak Ltd. or L. Light & Co.) was purified before use by fractional distillation under reduced pressure. The acetone used for Group I was an ordinary grade, purified by fractional distillation only. For Group II " Analar " acetone was boiled under reflux with solid potassium permanganate until no further decolorisation occurred. The solution was filtered, distilled, dried with anhydrous potassium carbonate, filtered again and fractionally distilled with precautions to exclude moisture (Vogel, 1956). Solutions of $\beta$-propiolactone were made up 2·5 per cent w/v every 2–3 weeks.

3-Bromopropionic acid (B.D.H.) was applied in a 2·5 per cent w/v solution in the redistilled acetone as used for Group I. All solutions were stored at 4° C.

### *Animal experiments*

The backs of inbred stock albino mice, as normally used in this department for carcinogenicity tests, were shaved before commencing treatment and at intervals thereafter as necessary. Hair growth was appreciably slowed during treatment with $\beta$-propiolactone but not with 3-bromopropionic acid.

*Group* I.—Twenty-five mice were treated with 0·3 ml. of the $\beta$-propiolactone solution in redistilled acetone on the back once weekly for 12 weeks, then twice weekly for a further 29 weeks. The 20 surviving mice were killed 5 weeks later. The mice yielded only one tumour, which was identified 41 weeks after commencing treatment as a squamous cell carcinoma.

*Group II.*—Forty-five mice were treated twice weekly for 60 weeks with 0·3 ml. of the solution of redistilled $\beta$-propiolactone in the specially purified acetone. Mice were killed during the experiment as necessitated by the development of tumours. Sections were stained (Ehrlich's haematoxylin and eosin ; Weigert's iron haematoxylin and van Gieson's stain) for histological examination by Dr. A. T. Spencer.

Macroscopic papillomas were found in one mouse after only 16 weeks of treatment, and in a further four mice after 20 weeks. Similar tumours appeared on other mice at various times up to 60 weeks, and generally proceeded to squamous cell carcinomas. Althogether 20 mice developed well-differentiated squamous cell carcinomas, showing varying degrees of infiltration of muscle, in the painted area of skin. In addition, one mouse developed a squamous cell papilloma, one a keratoacanthoma, and two mice showed hyperkeratosis and acanthosis of the skin with changes suggesting early carcinoma. Macroscopic papillomas on five other mice were not examined histologically owing to post-mortem changes. In two mice, killed at 36 and 51 weeks, squamous cell carcinomas developed on the snout while the treated area of skin remained free of tumours. Other findings were two mice with unidentified tumours on the eyelid, one with a spindle cell sarcoma in the painted area of skin, and one mouse with leukaemia. The remaining 11 mice died tumour-free or were killed because of poor condition after 24 to 57 weeks of treatment.

*Group III.*—Twenty-five mice were treated with the 3-bromopropionic acid solution once weekly for 6 weeks, then twice weekly for 29 weeks. Four mice died during this period, but no evidence of tumour production had been observed when the experiment was terminated 5 weeks later. Since the bromopropionic acid in these experiments was applied in the distilled and not in the specially purified acetone, a check has since been made which has shown that no detectable decomposition, with loss of bromide ion, occurred in this solvent after eleven weeks at room temperature.

*Further animal tests*

In view of the remarkable resistance of guinea-pigs to most carcinogens of the polycyclic hydrocarbon type, it was thought desirable to see if they would be equally resistant to one of the newer types of carcinogen. β-Propiolactone is therefore being applied twice weekly to shaved guinea-pig skin at concentrations of 2·5 and 5·0 per cent in the purified acetone as used in the mouse experiment (Group II).

One animal, which is white but not a true albino, showed a number of dark patches on the skin after only 16 weeks of treatment with the 5 per cent solution. One patch was removed and identified by Dr. D. J. Parish as a junctional melanoma. This early result is thought to be connected with skin damage which the animal received earlier in the course of fighting. Evidence of tumour production has not been seen on other guinea-pigs which have been treated for 37 weeks.

*Action of β-propiolactone on cysteine and cystine*

The course of these reactions was conveniently followed using a " Cambridge " Heyrovsky-pattern polarograph with photographic recording as in the albumin experiments already reported (Searle, 1961). For polarography the test solution (1·0 ml.) was added to the ammoniacal cobaltous buffer (10·0 ml.) to which was also added 0·25 ml. of 0·25 per cent gelatin solution. The gelatin suppressed the maximum at the $Co^{++}$-Co reduction but not the catalytic cysteine maximum, the height of which was measured from the level cobalt diffusion current to give a measure of the amount of unchanged cyst(e)ine in solution.

*Cysteine reaction*

To 100 mg. of cysteine hydrochloride dissolved in 10 ml. of water and just neutralised to bromothymol blue (pH range 6·0–7·6) was added 0·10 ml. of β-propiolactone (0·115 mg., 2·5 mol. proportions). 0·5 N NaOH was added at frequent intervals to counteract the acidity produced by hydrolysis of the lactone. After 7·5, 15, 30 and 60 minutes aliquots of solution were removed, diluted 100-fold with water, and polarographed in the cobaltous buffer solution from $-0 \cdot 9$ to $-2 \cdot 1$ v at a galvanometer sensitivity of 1/200.

For the reactions in weak acid and alkali bromothymol blue was replaced by bromocresol green (pH range 3·6–5·2) and phenolphthalein (8·3–10·0) respectively.

In neutral solution, a trace of cysteine was just detectable 30 minutes after adding the lactone but not after 60 minutes. In mildly alkaline solution all cysteine had disappeared by 7·5 minutes. In the acid range reaction occurred but was very slow, the height of the catalytic cysteine maximum being reduced by only 18 per cent after 30 minutes.

In each experiment the solution was finally made just alkaline to phenolphthalein and warmed to 60°, when some further reaction occurred in the acidic experiment. In no experiment was there any recovery of the catalytic wave, and there is therefore no evidence for formation of thioester in addition to S-2-carboxyethylcysteine.

*Cystine reaction*

Due to its insolubility cystine solutions containing only 0·76 mg. in 10 ml. were employed, but 0·10 ml. of β-propiolactone were added as before, i.e. a much greater excess. The solutions were added without dilution to the cobaltous buffer, and polarographed at a sensitivity of 1/500.

An unexpected finding was an increase in the height of the catalytic maximum on addition of β-propiolactone. In the experiment in neutral solution the maximum rose from 19·5 scale units at $-1 \cdot 85$ v to 24·5 units at $-1 \cdot 94$ v after 7·5 minutes, and then rose more slowly to become an inflection at 40 units at $-2 \cdot 1$ v after 60 minutes. A slight reduction in height occurred on warming to 60° in alkaline solution but the original maximum was not recovered. The changes observed in the mild alkali were almost identical with those in neutral solution. In mild acid, however, the cystine appeared completely unaffected by β-propiolactone.

Some polarograms obtained in the reactions of β-propiolactone with cystine and cysteine in neutral solution are illustrated in Fig. 2.

### DISCUSSION

In the only previous report on the carcinogenicity of β-propiolactone for mouse skin (Roe and Glendenning, 1956) papillomas began to appear on 5 out of 9 surviving mice after 27 weeks of weekly application of the lactone (2·5 per cent in " Analar " acetone). After 40 weeks, tumours on two of these mice became malignant and on two others were regarded as probably malignant. Tumours were obtained earlier in experiments when treatment for the first 5 weeks was with a 5 or 10 per cent β-propiolactone solution, which gave rise to ulceration and scabbing, and scarring appeared to have a tumour-promoting affect.

Our first series of mice treated with β-propiolactone only yielded one tumour. We attributed this result to the decomposition of a considerable proportion of the lactone by water still present in the acetone after fractional distillation. " Analar " acetone, which was used by Roe and Glendenning, is now stated to contain a maximum of 0·7 per cent of water, which is however sufficient to hydrolyse 2·8 per cent by weight of added β-propiolactone. When purifying this solvent we found it to reduce considerable amounts of potassium permanganate, suggesting the presence of alcoholic impurity which could also react with β-propiolactone. Destruction of lactone would probably be small, however, if the solution were freshly prepared before each application.

FIG. 2.—Superimposed tracings of polarograms obtained in reactions of β-propiolactone with cystine (A) and cysteine (B) in neutral solution. Polarograms recorded in Brdička's cobaltous buffer containing gelatin, using saturated calomel electrode. (I) Before addition of lactone; (II) 15 minutes and (III) 60 minutes after adding lactone. Galvanometer sensitivity 1/500 (A) or 1/200 (B). (The first wave at approximately 1·0 v represents the $Co^{++}$–Co reduction.)

When for Group II we used β-propiolactone in permanganate-treated, dried acetone, applied twice as frequently as in Roe and Glendenning's experiments, some papillomas occurred earlier than found by these authors, but on other animals papillomas took as long as 60 weeks to appear. Of the 34 mice of which stained sections were examined 20 had squamous cell carcinomas in the painted area, and of the original 45 mice only 11 were tumour-free at death. A tendency was noticed for tumours to occur somewhat earlier in mice with some skin damage, due to clipping of hair or to fighting, as has been observed with some other carcinogens.

The carcinogenicity of β-propiolactone for mouse skin is thus amply confirmed, though it is of course considerably less than that of the polycyclic hydrocarbon carcinogens which give a high yield of tumours when applied at less than one tenth of the dosage used here on a weight basis, or about one fortieth of the dose when allowance is made for the much smaller molecular weight of $\beta$-propiolactone. It would certainly seem advisable for human exposure to $\beta$-propiolactone itself to be as limited as possible.

Whether or not there is any carcinogenic risk attached to materials which have been sterilised with $\beta$-propiolactone, however, depends only on its breakdown products since all the lactone is destroyed in the process. Of these the major compounds would be 3-hydroxypropionic acid arising from hydrolysis and 3-chloropropionic acid from reaction with chloride ion, particularly in plasma.

Our first experiment appears to indicate that 3-hydroxypropionic acid is virtually devoid of carcinogenic activity, since in the solution with which these mice were painted the original lactone must have been largely hydrolysed to this acid. Dickens and Jones (1961) have also been unable to demonstrate any carcinogenic activity of hydrolysed $\beta$-propiolactone when tested in rats. While 3-chloropropionic acid has not, so far as is known, been tested for carcinogenic activity, the bromo-analogue was found inactive in the experiment reported above (Group III). The only other halogenated fatty acid tested appears to be iodoacetic acid, which Orr (1938) found to cause no changes not attributable to the solvent when he applied it to mouse skin as a 2 per cent solution in benzene.

It is possible that biological material treated with $\beta$-propiolactone would contain some S-2-carboxyethylcysteine. This compound was also tested for carcinogenicity by Dickens and Jones, who found a sarcoma in the only rat to survive 87 weeks of repeated injections of 0·5 mg. The significance of this result is difficult to assess at present.

It is difficult to correlate the observed effects of $\beta$-propiolactone on cysteine and cystine in protein combination with its action on the free amino acids. This is in part owing to the lack of exact knowledge concerning the origin of the catalytic double wave which is obtained when cyst(e)ine-containing proteins are polarographed in ammoniacal cobaltous buffer. It is usually assumed that only the second part of the double wave is directly due to the presence of cysteine and/or cystine in the protein. If this is so, the results of Searle (1961) indicate that cystine as well as cysteine in the protein can be attacked by $\beta$-propiolactone, since similar results were obtained with egg albumin and with bovine serum albumin, only the former of which contains appreciable cysteine in the reduced form. Moreover, the reaction was reversible, leading to the suggestion that a thioester was formed which became hydrolysed once more when the excess of lactone had been destroyed by the solvent.

The experiments with free cysteine, however, gave the results which would be expected in view of Dickens and Jones' isolation of S-2-carboxyethylcysteine in high yield, i.e. a complete irreversible destruction of the cysteine catalytic wave on addition of $\beta$-propiolactone. Cystine showed an increase in the catalytic wave due to some interaction which cannot at present be identified.

Some attempts were made during the present work to detect the formation of S-2-carboxyethylcysteine in $\beta$-propiolactone-treated albumins by two-dimensional paper chromatography of the desalted acid hydrolysates. This method did not give a definite result since glutamic acid, present in large amount, moved to an adjacent position on the paper with the solvent mixtures employed. It was confirmed that little if any destruction of S-2-carboxyethylcysteine would have occurred during hydrolysis.

We have thus no evidence that S-alkylation of cysteine can occur when the amino acid is in protein combination, or whether the ready S-alkylation of free cysteine has significance with regard to carcinogenesis.

SUMMARY

1. β-Propiolactone gave rise to papillomas and squamous cell carcinomas in a high proportion of mice when applied to the skin in carefully purified acetone.

2. 3-Bromopropionic acid showed no evidence of carcinogenicity for mouse skin.

3. The rapid reaction of β-propiolactone with cysteine, followed polarographically, was accelerated in mild alkali and retarded in mild acid.

4. Some form of interaction with cystine, also accelerated by mild alkali, was suggested by a rise in the polarographic catalytic maximum in cobaltous buffer.

5. Unlike the reaction of β-propiolactone with albumin cyst(e)ine groups, the changes observed with free cystine and cysteine were irreversible.

I am very grateful to Dr. A. T. Spencer for reporting on the tumour sections, and to Dr. D. L. Woodhouse for helpful discussions. The work was supported by the Birmingham Branch of the British Empire Cancer Campaign.

### REFERENCES

Dickens, F. and Jones, H. E. H.—(1961) *Brit. J. Cancer*, **15**, 85.

*Iidem* and Williamson, D. H.—(1956) *Rep. Brit. Emp. Cancer Campgn.*, **34**, 100.

Fieser, L. and Fieser, M.—(1956) "Organic Chemistry". New York (Reinhold), 3rd Edition, p. 323.

Hartman, F. W., LoGrippo, G. and Kelly, A. R.—(1954) *Amer. J. clin. Path.*, **24**, 339.

Orlans, E. S. and Jones, V. E.—(1958) *Nature, Lond.*, **182**, 1216.

Orr, J. W.—(1938) *J. Path. Bact.*, **46**, 495.

Rains, A. J. H., Crawford, N., Sharpe, S. H., Shrewsbury, J. F. D. and Barson, C. J.—(1956) *Lancet*, ii, 830.

Roe, F. J. C. and Glendenning, O. M.—(1956) *Brit. J. Cancer*, **10**, 357.

*Idem* and Salaman, M. H.—(1958) *Brit. med. J.*, ii, 942.

Schöberl, A. and Wagner, A.—(1956) *Z. physiol. Chem.*, **304**, 97.

Searle, C. E.—(1961) *Biochim. biophys. Acta*, **52**, 579.

Smith, H. H. and Srb, A. M.—(1951) *Science*, **114**, 490.

Vogel, A. I.—(1956) "Practical Organic Chemistry". London (Longmans), 3rd Edition, p. 171.

Walpole, A. L., Roberts, D. C., Rose, F. L., Hendry, J. A. and Homer, R. F.—(1954) *Brit. J. Pharmacol.*, **9**, 306.